# Early life vitamin D depletion and mechanical loading determine methylation changes in the *RUNX2*, *RXRA*, and *osterix* promoters in mice

**DOI:** 10.1186/s12263-022-00711-0

**Published:** 2022-05-26

**Authors:** Nevena Krstic, Nick Bishop, Beth Curtis, Cyrus Cooper, Nick Harvey, Karen Lilycrop, Robert Murray, Robert Owen, Gwen Reilly, Tim Skerry, Steph Borg

**Affiliations:** 1 Biological Sciences and NIHR Southampton Biomedical Research Centre, University of Southampton, Southampton, UK; 2 Department of Oncology & Metabolism, University of Sheffield Medical School, Beech Hill Road, Sheffield, S10 2RX UK; 3 MRC Lifecourse Epidemiology Unit, Southampton General Hospital, University of Southampton, Southampton, UK; 4 Rheumatology Department, University Hospital Southampton NHS Foundation Trust, Southampton, UK; 5 NIHR Oxford Biomedical Research Unit, University of Oxford, Oxford, UK; 6 Department of Materials Science and Engineering, Kroto Research Institute, The University of Sheffield, Sheffield, UK; 7 INSIGNEO Institute for in silico Medicine, The University of Sheffield, Sheffield, UK; 8 Regenerative Medicine and Cellular Therapies, School of Pharmacy, University of Nottingham, Nottingham, UK

**Keywords:** Vitamin D, Mechanical loading, Bone, Methylation, RXRA, Osterix, Epigenetics

## Abstract

**Background:**

Early life vitamin D exposure is linked to later skeletal health with maternal vitamin D status in pregnancy associated with neonatal bone mass. The MAVIDOS study has demonstrated that vitamin D supplementation leads to reduced RXRA DNA methylation. Mice exposed to early life vitamin D deficiency have reduced bone mass and bone accrual in response to mechanical loading. Using the tibiae of these mice, we have examined the effect of diet and mechanical loading on the DNA methylation of promoters of genetic loci important for bone growth and development and their association with bone strength.

**Results:**

Mechanical loading of mouse tibiae leads to a reduction of RXRA DNA methylation. Early life vitamin D deficiency is associated with altered methylation of *osterix* and *Runx2* in these bones. Tibia strength was also demonstrated to be associated with a change in DNA methylation status in CpGs of the vitamin D receptor (VDR), ostrix, and RXRA genes.

**Conclusions:**

We have shown for the first time that mechanical loading of bone and early life vitamin D deficiency leads to changes in the epigenome of this tissue in key genes in the vitamin D and osteoblast differentiation pathway.

## Background

Early life exposures have been hypothesised to lead to long-term changes in adult health [1]. Suboptimal early life exposures including maternal nutrition can lead to long-term adverse health effects [2], with poor intrauterine growth, a predictor of later osteoporosis and fracture risk [3, 4]. There are both observational and interventional studies that show the effects of altered vitamin D exposure during pregnancy on bone mass accrual [4–9], although the mechanisms that are in play are incompletely understood.

One mechanism by which early life environment has been suggested to influence later phenotype is through the altered epigenetic regulation of genes. Epigenetic mechanisms induce heritable changes in gene expression without a change in the gene sequence. Epigenetic mechanisms include DNA methylation which can affect the accessibility of the DNA to the transcriptional machinery [10]. In general, hypermethylation of the promoter region is associated with gene silencing and hypomethylation with gene activity. There is now substantial evidence from animal models that the early life environment can affect DNA methylation, with changes for instance in maternal diet associated with changes in DNA methylation and expression of key metabolic genes and the metabolic processes they control in the offspring [11]. Maternal undernutrition, overnutrition, low-protein intake, and micronutrient status during pregnancy have all been shown to influence the distribution of methyl marks in offspring [12–17]. Similar observations have been observed in humans, with famine exposure in utero linked to altered DNA methylation in the adult offspring [18, 19], suggesting that early life environment can induce persistent epigenetic changes in the offspring.

To date, very few studies have examined the effect of vitamin D supplementation on DNA methylation as a potential mechanism by which vitamin D supplementation may affect bone accrual. Altered RXRA methylation has previously been shown to be predictive of bone health in later childhood in humans [20]. Altered methylation at ten previously identified CpG methylation sites in the *RXRA* locus has been observed in umbilical cord tissue stored at birth from the MAVIDOS study (multicentre, double-blind randomised, placebo-controlled trial of vitamin D 1000 IU/day from 14-week gestation to delivery) in the maternal supplementation group. This study found a positive effect of maternal vitamin D supplementation during pregnancy on neonatal bone mass for winter births (when background 25(OH)D-vitamin D concentrations are lowest). This suggests vitamin D may affect bone development accrual through the epigenetic of regulation of RXRA and related pathways [21, 22]. Differential associations between *RXRA* methylation at birth and offspring bone mass at birth and in later childhood suggest the possibility that other environmental stimuli which affect bone outcomes, for example, mechanical loading, might also modify the epigenetic regulation of RXRA and genes related to bone development.

We have also assessed the response to mechanical stimulation using whole-body vibration (WBV) of a subgroup of the MAVIDOS cohort children. We found that those children whose mothers received vitamin D supplementation had a greater response to WBV as assessed by a rise in the circulating bone formation marker P1NP compared to the children whose mothers received placebo [23].

We previously created a murine model in which pregnant dams were either vitamin D deficient or replete, and their offspring moved to a vitamin D-replete diet at weaning. Tibias of the offspring were mechanically loaded, and offspring of vitamin D-deplete mice showed a lower bone mass in the non-loaded limb in the younger, skeletally immature, age group and reduced bone mass accrual in response to loading in both the growing and mature skeleton [24].

Taken together, both the human and preclinical data suggest that epigenetic processes may mediate the effect of early life environment during the in utero and young postnatal period whilst there is still plasticity in the genome. The accrual of bone mass during skeletal development is clearly complex and multifactorial. We have focused our attention on two key regulators of osteoblastic differentiation — *Osterix and Runx2* — as well as the critical components on the vitamin D pathway mediating the response to administered vitamin D, namely the vitamin D receptor (VDR) and retinoic acid receptor (RXRA). Together, they comprise the machinery that transduces the nuclear signalling of vitamin D. Runx2 has been shown to induce differentiation of MSCs into immature osteoblasts, whereas Osterix is involved in the differentiation of pre-osteoblasts into mature osteoblasts and osteocytes [25].

We therefore hypothesised that we would be able to demonstrate epigenetic alterations in key elements of the vitamin D metabolism pathway — *RXRA* and *VDR* — and in genes regulating osteoblastic differentiation — *Runx2* and *Osterix* [12, 26] as a consequence of early life vitamin D depletion in our preclinical model and in response to loading, and that those changes would be associated with altered bone biomechanical properties.

Within this manuscript, we utilise data from our previously published work examining the effect of early life vitamin D deficiency on the bones of these mice. The data for bone strength (stiffness and ultimate force), as measured by 3-point bending on the harvested post-mortem bones, is newly evaluated within linear regression analysis of the epigenetic data newly presented here. This was possible as the same bones analysed for 3-point bending were used for the DNA extraction and subsequent pyrosequencing [24]. Here, we present our analyses of the effect of diet and loading on the methylation status of the promoters in genes crucial in the vitamin D pathway and osteoblast differentiation in mouse tibias subjected to mechanical loading and later post-mortem biomechanical testing.

## Results

### Vitamin D status

At weaning, all mice, irrespective of early life dietary exposure, were weaned onto the control vitamin D-containing diet. All animals were vitamin D replete when mechanically loaded, and we have previously reported this data [24].

The effect of early life vitamin D deficiency on the DNA methylation of Runx2, Osterix, VDR, and RXRA, genes known to play essential roles in bone formation, was assessed. Pyrosequencing primers were designed across the proximal promoter regions of these genes (Fig. 4).

### Mechanical loading alters DNA methylation status in mice tibiae

**Fig. 1:**
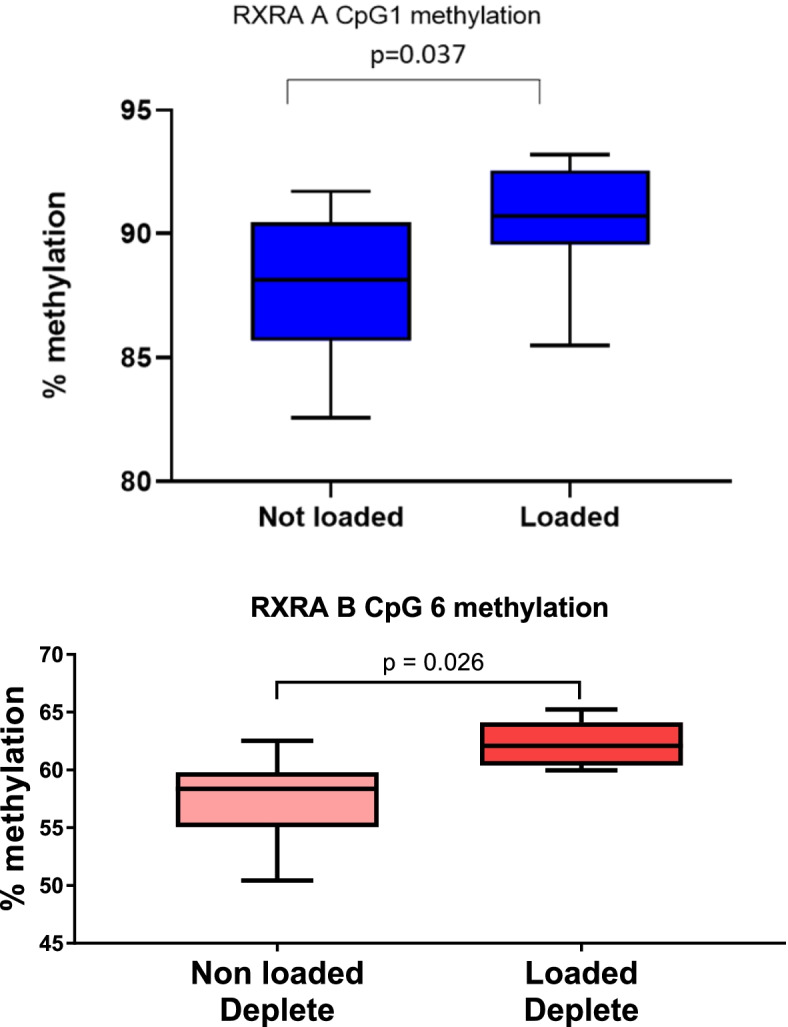
Effect of loading on (**a**) RXRA A CpG1 site methylation irrespective of early life diet exposure (*n* = 12/group) (**b**) RXRA B CpG6 site methylation when exposed to early life vitamin D deficiency (*n* = 6/group)

DNA methylation was assessed in response to loading irrespective of prenatal and early life vitamin D depletion/repletion. Mice had lower levels of DNA methylation at the *RXRA* CpG1 site in the non-loaded as opposed to loaded limbs (*p* = 0.0373; difference −2.47% 95% *CI* −4.79, −0.16; non-loaded *n* = 11/group; loaded = 12/group) (Fig. 1a). There were no significant differences in methylation at individual CpGs upstream of the *VDR*, *Osterix*, or *Runx2* promoters between the groups.

The mice were analysed with respect to early life vitamin D deficiency or sufficiency. We found that mechanical loading was associated with lower levels of DNA methylation at the RXRA B CpG 6 site in the non-loaded limb compared to the loaded limb in those exposed to early life deficiency (*p* = 0.026, mean difference = −4.7%, 95% *CI*: −8.80, −0.69; *n* = 6/group) (Fig. 1b). There were no significant differences in methylation status in the other genes examined in response to mechanical loading.

### Early life vitamin D deficiency is associated with altered offspring DNA methylation in mouse tibiae

**Fig. 2:**
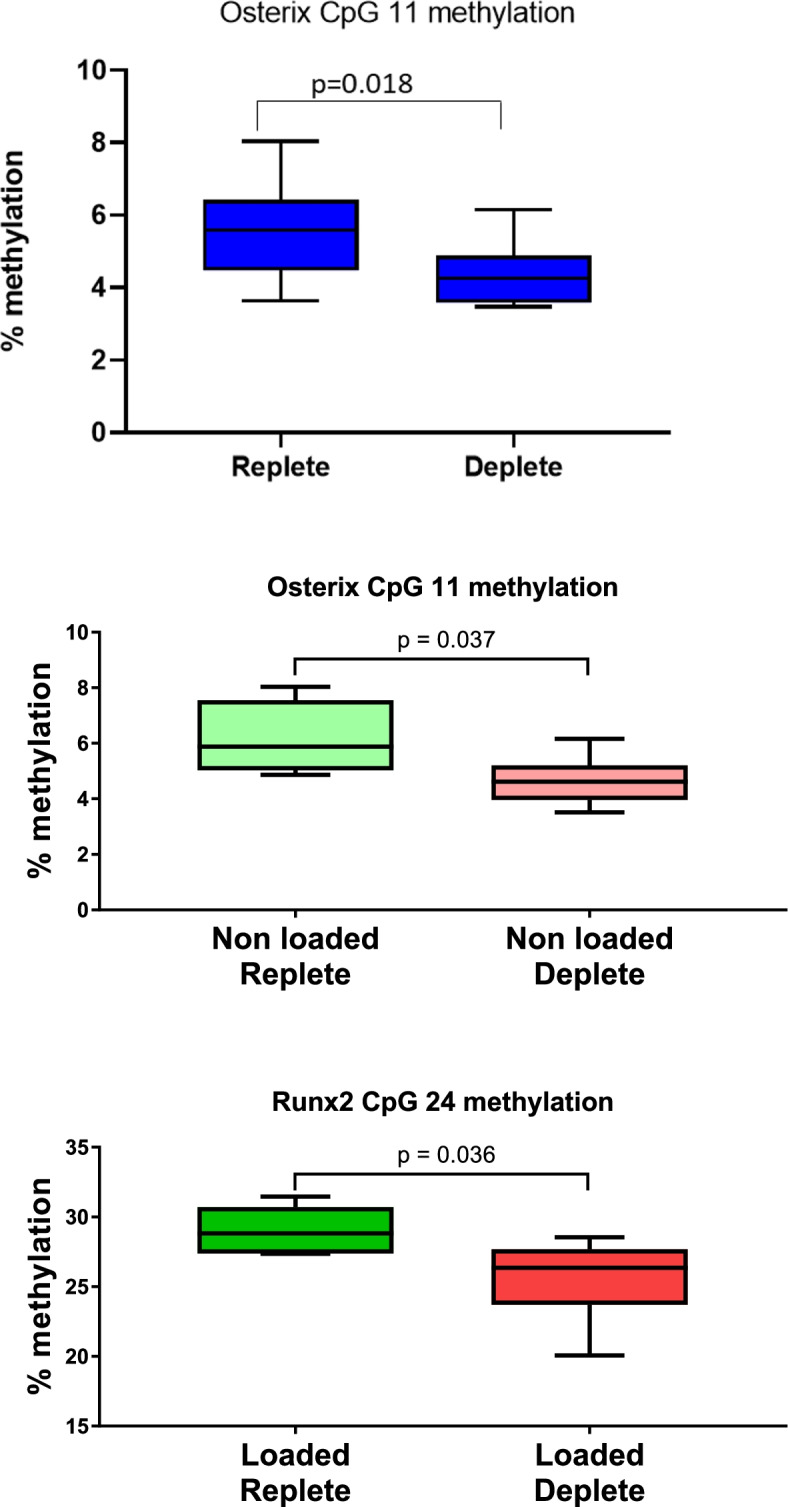
Effect of early life vitamin D deficiency on (**a**) osterix CpG11 site methylation irrespective of loaded (*n* = 12/group) and (**b**) in non-loaded tibiae (*n* = 6/group) and (**c**) Runx2 CpG methylation in loaded tibiae (*n* = 6/group)

DNA methylation changes in response to prenatal and early life vitamin D depletion irrespective of loading were first investigated. Methylation of *Osterix* CpG 11 was reduced in the tibiae of all offspring mice exposed to an early life diet deficient in vitamin D compared to the tibiae of offspring mice exposed only to the control diet (*p* = 0.0184, mean difference = 1.16%, 95% *CI* 0.22, 2.10; *n* = 12/group) (Fig. 2a). There were no significant differences in methylation at individual CpGs upstream of the *RXRA*, *VDR*, or *Runx2* promoters between the diet groups.

We next assessed epigenetic changes in response to prenatal and early life vitamin D depletion in the absence of loading. Methylation of *Osterix* CpG 11 was reduced in the non-loaded tibiae of offspring mice exposed to a diet deficient in vitamin D compared to the non-loaded tibiae of offspring mice exposed to the control diet (*p* = 0.031, mean difference = 1.50, 95% *CI* 0.12, 2.96; *n* = 6/group) (Fig. 2b). There were no significant differences in methylation at individual CpGs upstream of the *RXRA*, *VDR*, or *Runx2* promoters between the diet groups.

We then assessed epigenetic changes in response to prenatal and early life vitamin D depletion in the presence of loading. Methylation of *Runx2* CpG 24 was reduced in the loaded tibiae of offspring mice exposed to a diet deficient in vitamin D compared to the loaded tibiae of offspring mice exposed to the control diet (*p* = 0.036, mean difference = 3.4%, 95% *CI* 0.27, 6.61; *n* = 6/group) (Fig. 2c). There were no significant differences in methylation at individual CpGs upstream of the *RXRA*, *VDR*, or *Osterix* promoters between the treatment groups.

### Extrinsic tibia strength is associated with changes in DNA methylation status

There was a positive association between tibial stiffness and DNA methylation of *VDR* CpG 4 and CpG 5 in all the bones examined irrespective of diet and loading (*b* = 0.315, *r*^2^ = 0.776, *p* = 0.0008, *r*^2^ = 0.746, *b* = 0.261, *p* = 0.031, respectively; *n* = 24).

For non-loaded limbs, DNA methylation status of *VDR* CpG 1, CpG 4, and CpG 5 was positively associated with tibial stiffness irrespective of diet (*b* = 0.465, *p* < 0 = 0.03; *b* = 0.82, *p* = 0.0039; *b* = 0.66, *p* = 0.04, respectively; *n* = 12).

For loaded limbs, DNA methylation status of *VDR* CpG 9 was negatively associated with tibial stiffness irrespective of diet (*b* = −1.44, *p* = 0.0362; *n* = 12).

The percentage change in DNA methylation status in *RXRA* A CpG 4 and *Osterix* CpG 11 in response to loading was negatively associated with the percentage change in stiffness following mechanical loading irrespective of diet (*b* = −2.03, *p* = 0.02; *b* = 0.04, *p* = 0.0088) (Fig. 2).

The percentage change in DNA methylation status of *Osterix* CpG 17 was negatively associated with the percentage change in ultimate force following mechanical loading irrespective of diet (*b* = −0.5, *p* = 0.019).

## Discussion

We have previously shown in this group of mice that early life vitamin D deficiency led to lower bone mass and reduced bone mass accrual in response to mechanical loading [24]. We are now able to show that as well as the phenotype being altered; the epigenome is altered in the skeleton of the adult offspring exposed to early life vitamin D deficiency and, separately, in response to mechanical stimulation. This suggests that the early life environment can have long-term consequences for the skeleton that persist into adulthood.

We found alterations in DNA methylation in the promoters of four genes whose functions are important for skeletal development and bone accrual. Early life dietary deficiency of vitamin D was associated with altered methylation in *Osterix* (non-loaded bone) and *Runx2* (loaded bone). Thus, we have shown, for the first time, that mechanical loading of bone can alter DNA methylation; this was seen in *RXRA* A CpG 1 irrespective of early life vitamin D depletion and in *RXRA* B CpG 6 in mice who had had early life depletion.

Methylation status in *VDR* CpG 4 and 5 was associated with tibial stiffness irrespective of diet or loading; this association was maintained for non-loaded limbs irrespective of dietary exposure, where *VDR* CpG 1 methylation status was also associated with tibial stiffness. Change in percentage methylation status in response to loading was associated with change in stiffness for two sites: *RXRA* A CpG 4 and *Osterix* CpG 11. The only association with change in ultimate force in response to loading was the change in methylation status of *Osterix* CpG 17.

DNA methylation was measured upstream of the transcriptional start site of four genes: *RXRA*, *VDR*, *Runx2*, and *Osterix*. *RXRA* and *VDR* play an important role within the vitamin D pathway; their heterodimerisation is key to mediating their downstream transcriptional regulatory activity through targeting of specific vitamin D response elements in the promoter regions of many genes. The vitamin D receptor is widely distributed within different tissues, and effects on a broad range of biological processes have been linked to current and prior vitamin D status [27]. *Runx2* and *Osterix* are transcription factors which play critical roles in osteoblast differentiation. Runx2 is a primary transcription factor and one of the earliest markers of osteoblastogenesis; it lies upstream of *Osterix* in the osteoblast differentiation pathway [28].

A prenatal vitamin D-deficient diet, either irrespective of loading or in the absence of loading, was associated with lower *Osterix* CpG 11 methylation compared to the control diet. The *Osterix* CpGs of interest are located 369 bp upstream of the *Osterix* transcription start site (TSS) within the promoter region. The functional importance of these CpGs has not been investigated previously; however, methylation in promoter regions is usually associated with gene repression [29]. Reduced *Osterix* promoter methylation in mice born to vitamin D-deficient mothers might thus be associated with increased *Osterix* expression which would be expected to promote osteoblastic differentiation. Early life vitamin D deficiency did not alter trabecular parameters in non-loaded bones of these mice at age 18 weeks, but there was an increase in the ratio of mineralisation surface to tibial bone surface at the medial and lateral areas of the tibia and increased cortical porosity compared to the control mice suggesting increased bone turnover at those sites [24]. This could be part of a compensatory mechanism in response to low maternal vitamin D during pregnancy to maintain skeletal homeostasis.

In the loaded limbs, mice exposed to a prenatal vitamin D-deficient diet had lower *Runx2* methylation at the CpG 24 site compared to control mice. The *Runx2* CpGs are located over 2-kbp upstream of the *Runx2* TSS, and Ensembl maps this region as part of the *Runx2* promoter. Wakitani et al. found that there was an inverse association between DNA methylation of *Runx2* CpG-2101 and *Runx2* gene expression [30]. This CpG site is 40 bp away from our CpGs of interest, so it is likely that similar associations would be shown. This would suggest that lower methylation is likely to be associated with increased *Runx2* gene expression.

Studies in mice have shown that *Runx2* overexpression can promote osteoclast differentiation through *RANKL* (expressed by both osteoblasts and osteocytes), and that loss of *Runx2* expression leads to reduced/absent RANKL likely due to maturational arrest of osteoblasts, with normal levels of OPG. Therefore, a decrease in *Runx2* methylation could be linked to an increase in *Runx2* expression, with increased osteoclast differentiation through osteoblast-expressed *RANKL*. Conversely, *Runx2* overexpression also inhibits osteoblastic differentiation [31, 32]. Reduced osteoblast differentiation would likely reduce bone formation; coupled with increased osteoclastic resorption, this might contribute to our previous findings of lower bone mass and reduced bone accrual in response to mechanical loading.

Irrespective of prenatal diet, loading induced epigenetic changes at the *RXRA A* CpG1 site, with reduced methylation in the non-loaded as opposed to loaded limbs. For mice exposed to early life vitamin D deficiency, mechanical loading was linked to higher *RXRA* B CpG 6 methylation compared to the non-loaded limb. These *RXRA* CpGs are located 390-bp upstream of the *RXRA* TSS, within the promoter region. In the mice exposed to prenatal vitamin D deficiency, mechanical loading significantly improved trabecular and cortical parameters (but to a lower extent than in those who were vitamin D replete), but there was a decrease in the ratio of mineralisation surface to bone surface at the lateral part of the tibia [24]. *RXRA* has been shown to play a role in osteoclast differentiation, and a study of the loss of *RXRA* function in haematopoietic cells resulted in giant non-resorbing osteoclasts and increased bone mass [33]. In the MAVIDOS trial, we have shown that maternal cholecalciferol supplementation during pregnancy is associated with lower *RXRA* CpG methylation in umbilical cord tissue compared to cord tissue from placebo-supplemented mothers [21]. Moreover, studies within the Southampton Women’s Study cohort have shown that there is an inverse association between *RXRA* CpG methylation and bone measures in childhood [20]. Our findings suggest that *RXRA* CpG methylation in relation to vitamin D depletion may exert its effect on bone parameters through reduced bone formation.

By comparing DNA methylation in loaded and non-loaded bones, we have shown for the first time that mechanical tibial loading alters DNA methylation (of *RXRA*) from the same bone tissue. The mechanostat describes the mechanism by which structural changes in bone architecture occur in response to mechanical loading. We demonstrate here that DNA methylation plays a role in this mechanism by either initiating a change in expression or consolidating a change in expression mediated by a change in histone modification. Moreover, as DNA methylation is a relatively stable mark, this also suggests a mechanism by which bone loading in early life may induce persistent effects at later time points through the life course.

We have previously demonstrated in these mice that the values for stiffness and ultimate force (measure of extrinsic bone strength) of their tibiae were similar. That mechanical loading did not, however, increase bone stiffness in the early life-deficient mice unlike the control group. The force required to break the bone (ultimate force) in both groups of mice increased in response to mechanical loads but by a lesser amount in the early life-deficient mice [24]. DNA methylation of *VDR* in non-loaded limbs is positively associated with tibial stiffness and negatively associated in loaded limbs. The *VDR* CpGs are located 26 bp from the *VDR* TSS, within the promoter, and therefore are likely to lead to a decrease in *VDR* expression. The ability of epigenome to change in response to the external stimulus of mechanical loading in adult life is a novel finding.

This study focused on CpGs within the proximal promoters; it would be interesting to look across the whole promoter region and upstream enhancer regions. A further potential study limitation is in CpGs of interest, we are seeing relatively low percentage changes in methylation (< 5%), and it is unknown what such differences mean. We presume this to reflect the number of cells expressing the gene in a tissue and so may affect function. Genomic DNA isolated from whole blood of individuals who were in utero during the Dutch Hunger Winter compared to unexposed same-sex siblings has shown hypomethylation of the differentially methylated regions of the imprinted IGF2 gene with a change of 5.2% [18]. The adult offspring of mothers in the Motherwell cohort — pregnant women encouraged to eat 0.45 kg red meat daily and low carbohydrate foods — was recruited to examine their epigenome. Low levels of methylation change were identified at similar levels to our study [34]. A further limitation is that we have no cell type correction data available. Tibial bone consists of many cell types, and methylation patterns are known to be tissue and cell type specific. A fourth limitation is that the mice were loaded twice, during their pubertal growth between weeks 8 and 10 and during adult growth in weeks 16–18. Ideally, DNA methylation would have been measured in mice that were only loaded in adulthood. Furthermore, no other studies have looked at the effects of two periods of loading.

## Conclusion

We have shown for the first time changes in the epigenome in adult mice that following mechanical loading of bone with differential methylation are present in the epigenome of key genes in the vitamin D and osteoblast differentiation pathways.

We have also demonstrated a series of changes in methylation in response to early life vitamin D depletion with and without the effects of mechanical loading. These give support to the hypothesis that early life events can have long-lasting consequences for skeletal health and further support our view that vitamin D supplementation during pregnancy and early life is a crucial factor that can be modified for the benefit of future generations.

## Methods

### Animals

**Fig. 3:**
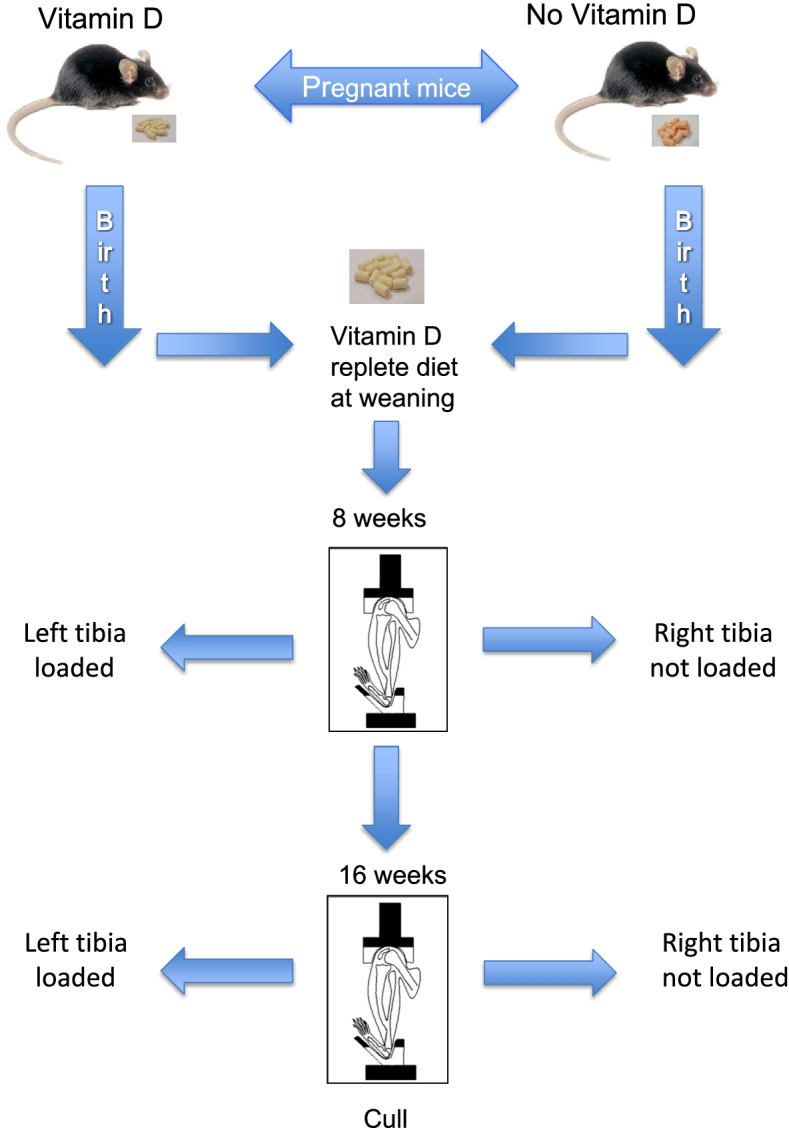
Experimental design showing creation of the murine model of early life vitamin D deficiency. All mice underwent 2 episodes of mechanical loading of their left tibia at age 8 and 16 weeks. The right tibia was not mechanically loaded and provided an internal control. Following the second episode of loading at 16 weeks of age, mice were culled (aged 18 weeks), and both tibias stored frozen until 3-point bend testing and subsequent DNA extraction from each of the whole bones (*n* = 6 mice/*n* = 12 bones per dietary group). Adapted from Borg et al. [24].

The murine model has been described in detail previously (Fig. 3) [24]. Four-week-old C57BL/6 (Charles River, Kent, UK) female mice were purchased and housed at the University of Sheffield Biological Services Unit and had access to food and water ad libitum. All mice were housed in a single room with a 12-h light/dark cycle at 22 °C under UV-filtered fluorescent lighting.

Female mice were fed a vitamin D-supplemented diet (1000 units/kg) or vitamin D-free diet based on AIN93G (Research Diets Inc., USA) diet from 4 weeks of age. Mice were randomly allocated to either vitamin D replete (1000 units/kg) or deplete diet. At 10 weeks of age, female mice were mated with healthy control adult males; dams remained on their respective diets throughout gestation until pup weaning. Female offspring remained with their dams until weaning at day 22 whereupon they were weaned onto the vitamin D-replete diet (*n* = 24) (male pups not included in this protocol). All animals were supplemented with 2 mmol/l Ca^2+^ in their drinking water.

This study was carried out as per regulations under the Animals (Scientific Procedures) Act 1986. The study was approved by the local committee of the University of Sheffield that oversees animal use within the institution. Loading experiments were performed under isoflurane anaesthesia. All efforts were made to minimise animal suffering.

### Mechanical loading

A noninvasive method of tibial loading was used at ages 8 and 16 weeks as previously reported [24]. The peak load of 11 N was selected as this is known to induce an osteogenic response in female C57BL/6 mice [35–37]. A 10.5-N dynamic load was superimposed onto a 0.5 N preload at a rate of 160,000 N/s. Forty trapezoidal-waveform load cycles (0.2 s hold at 11 N) with a 10-s interval between each cycle were applied to each mouse’s left tibia 3 times per week for 2 weeks.

Mice were euthanised on day 15 (where day 1 was the first of the loading cycle following the second episode of loading at age 16 weeks. Both tibias were dissected and frozen wrapped in PBS-soaked gauze at −20 °C. The contralateral non-loaded limb (right tibia) was treated as an internal control for loading (the functional adaptation of the bone is confined to the loaded limb) [38]

### Bone mechanical testing

Bones previously stored at −20 °C in PBS-soaked gauze were placed in PBS at 4 °C overnight prior to testing at room temperature. Samples were subjected to three-point bending analysis using a Bose ElectroForce 3200 mechanical testing machine with a 450 N load cell as previously described [24]. Briefly, rounded supports and press head were used with a constant span support distance of 6 mm for all tibias; bones were positioned with the press head contacting the midshaft. A preload of 0.5 N was applied to the bone before compressing at a constant force of 0.25 N/s until failure. From the force-displacement curve, stiffness (n/mm) was calculated from the linear elastic region and breaking force from the load at failure.

### Genomic DNA extraction

Following biomechanical testing, bones were again stored frozen at −20 °C wrapped in PBS-soaked gauze. DNA was extracted from tibiae using the DNeasy Blood and Tissue Kit as per the manufacturer’s instructions. DNA was stored at 4 °C for short-term use and at −20 °C for longer-term storage.

### Sodium bisulphite pyrosequencing

**Fig. 4:**
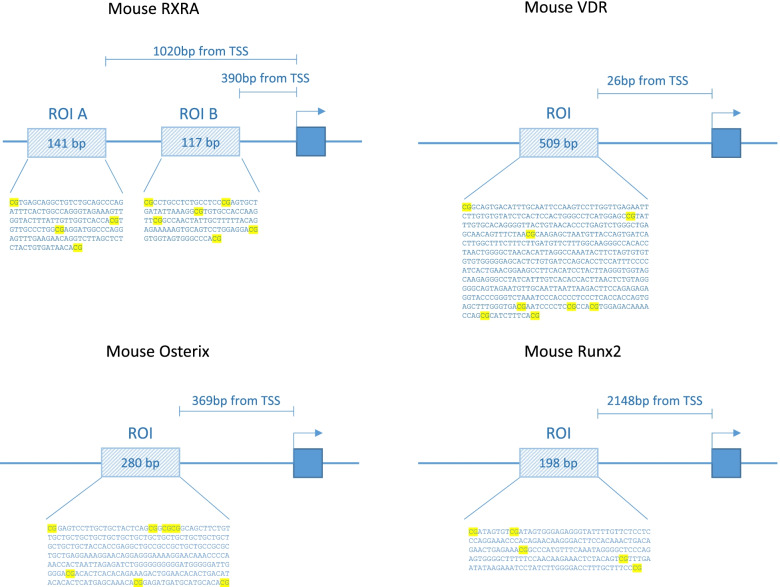
Location of the CpGs of interest with respect to the gene transcriptional start site (TSS). CpGs of interest are highlighted in yellow. ROI, region of interest

%DNA methylation of specific CpGs upstream of the promotors of the 4 examined genes (*RXRA*, *VDR*, *Runx2*, and *Osterix*) (Fig. 4, Table 1) was measured in the loaded and non-loaded tibiae using sodium bisulphite pyrosequencing. Briefly, murine DNA was bisulphite converted using the EZ DNA Methylation Gold bisulphite conversion kit (Zymo Research, #D5007) as per the manufacturer’s instructions. A total of 2 ul of bisulphite-converted DNA were used in subsequent PCR reactions using HotStarTaq (Qiagen #203205) and PCR primers for *RXRA*, *VDR*, *Runx2*, and *Osterix*, with the reverse primer containing a biotin tag, as per manufacturer’s instructions. DNA methylation was measured using the PyroMark pyrosequencer (PyroMark MD; QIAGEN, Hilden, Germany; https://www.qiagen.com/fi/resources/technologies/pyrosequencing-resource-center/technology-overview/).

**Table 1:** Pyrosequencing amplicons and sequencing primers

Gene	Amplicon		Sequencing primer	
**Mouse RXRA**	**RXRA A 1–4 60 °C**	F: GGTTGTGAGGGGGTAGAATTA R: CCCCACAAACCTTTCCTAATCC	**RXRA A 1 s** **RXRA A 2–3 s** **RXRA A 4 s**	GAGATTATAAGTTTTATTTTAGGGA TGTTTTTTAAATTTTTGGGTTATT TGAAATTTGGGTTGTAGAT
	**RXRA B 1–6 57 °C**	F: AGAGGGGTTTATTAAAGTTTATAGGT R: AATTTCTCTATATAACCCTAACTATCCT	**RXRA B 1–2 s** **RXRA B 3–4 s** **RXRA B 5–6 s**	TTTTAAGATAAAAAAGAAAGT TTTTTTTTGTAAAAAGTAATAGT TGGTGGTATAAGTTTTTAATATTAG
**Mouse Runx2**	**Runx2 24–28 57 °C**	F: ATGGAGTGGTGGTAATTAAATAGT R: ATAACTTACTTTCATTACCCCTCATT	**Runx2 25–24 S** **Runx2 26 S** **Runx2 27 S** **Runx2 28 S**	GGGAGGAGAATAAAATATTTTT GGGAGTTTTTATTTGAAATATG GGTTTTTAAGATAGGATTTTTTATA AGTGGTGGTAATTAAATAGTA
**Mouse Osterix**	**Osterix 8–18 54 °C**	F: GTTTTTATGTGGGTAGTAGAGAGT R: ATCCAATCCTACAATCCTACTCTTAA	**Osx 11–8 S** **Osx 17 S** **Osx 19–18 S**	AGTAGTAGTAGTAGTAGTAATAGA GTTTTAGTTTTTTTGTGTGAG ATGTGGGTAGTAGAGAGTA
**Mouse VDR**	**VDR 1–6 57 °C**	F: TGTTATATTATTTAATTTTGTAGGGGGTAG R: CCTCATAAAAACCAACTTACTTACTCT	**VDR 1–2 S** **VDR 3–5 S** **VDR 6 S**	TTTAGTTAAGTGGAGATAAAATTAG AGTGAGTTTTGGGTG AAGATTTTTAGAGAGAGGTA
	**VDR 8–10 60 °C**	F: GGTGTGGTTTTTGTTAAAGAATATTAAGA R: CACTCCCAAATTTCTAAACTAACCTTAA	**VDR 8 S** **VDR 9 S** **VDR 10 s**	AGTTAAGTGATTATTGGTAATAT GGTGTTATAGTAATTTTTGTGTAT AGGATTTGGAATTGTAAATG

Pyrosequencing primers were designed to measure the DNA methylation status of CpGs sites within the promoter regions of RXRA, VDR, and Osterix, where sequence context permitted the efficient binding of primers. For Runx2, DNA methylation was measured across CpG sites −2484 to −2148 bp upstream of the *Runx2* promoter, as this was a region previously identified to be differentially methylated [30].

### Statistical analyses

Independent *t*-tests compared %DNA methylation between the non-loaded and loaded limbs of mice exposed to a control or prenatal vitamin D-deficient diet. To determine whether there were any associations between DNA methylation and tibial stiffness or ultimate force, linear regression analyses were adjusted for the treatment group and for mechanical loading.

## Data Availability

The datasets analysed during the current study are available from the corresponding author on reasonable request.

## Abbreviations

ROI: Region of interest
RUNX2: RUNX family transcription factor 2
RXRA: Retinoid X receptor alpha
TSS: Transcriptional start site
WBV: Whole-body vibration
VDR: Vitamin D receptor
